# COVID-19: the relationship between perceptions of risk and behaviours during lockdown

**DOI:** 10.1007/s10389-021-01543-9

**Published:** 2021-05-13

**Authors:** Richard Brown, Lynne Coventry, Gillian Pepper

**Affiliations:** Psychology Department, Northumbria University, Northumberland Building, College Street, Newcastle Upon Tyne, NE1 8ST UK

**Keywords:** COVID-19, Risk perceptions, Health behaviours, Compliance, Mortality risk

## Abstract

**Aim:**

Understanding COVID-19 risk perceptions and their impact on behaviour can improve the effectiveness of public health strategies. Prior evidence suggests that, when people perceive uncontrollable risks to their health, they are less likely to engage in healthful behaviour. This article aims to understand the extent to which COVID-19 is perceived as an uncontrollable risk, and to assess whether this perceived risk is associated with health behaviour.

**Subject and methods:**

We surveyed a nationally representative sample of 496 participants during the first UK lockdown. We assessed perceptions of COVID-19-related risk, self-reported adherence to infection control measures recommended by the UK Government, and general health behaviours. We predicted that increased perceived extrinsic mortality risk (the portion of mortality risk perceived to be uncontrollable) would disincentivise healthy behaviour.

**Results:**

Perceived threat to life was the most consistent predictor of reported adherence to infection control measures. Perceived extrinsic mortality risk was found to have increased due to the pandemic, and was associated with lower reported adherence to Government advice on diet, physical activity, and smoking.

**Conclusions:**

Our findings suggest that health messages that highlight threat to life may be effective in increasing adherence to infection control, but may also lead to a reduction in health-promoting behaviours. We suggest that messages that highlight threat to life should be accompanied by statements of efficacy. Further, messages evoking feelings of concern for others may be effective in promoting compliance with anti-infection measures, without the potential for the unwelcome side-effect of discouraging healthy behaviour.

**Supplementary Information:**

The online version contains supplementary material available at 10.1007/s10389-021-01543-9.

## Introduction

The COVID-19 pandemic continues to have a devastating impact on countless lives across the globe. At the time of writing (February 2021), the World Health Organisation (2021) reports that over 102.1 million cases of COVID-19 have been registered, resulting in over 2.2 million COVID-19 related deaths. Studying perceptions of risk during the pandemic can develop our understanding of the psychological response to the threat of COVID-19 and help to provide effective public health strategies for the future (Dryhurst et al. 2020).

A perceived lack of control over risk can have consequences for behaviour. The Uncontrollable Mortality Risk Hypothesis predicts that people who believe they are more likely to die due to factors beyond their control should be less motivated to engage in healthy behaviours. Those who are exposed to uncontrollable risks remain relatively less likely to live to enjoy the rewards of healthy living, despite their health efforts. Therefore, resources (time, energy, and money) invested in future health, which could be directed elsewhere, are more likely to go to waste, which disincentivises healthful behaviour. This hypothesis has been supported by studies that show both observational and experimental effects of perceived uncontrollable (extrinsic) mortality risk on health behaviour (Pepper and Nettle 2014a, b, c, 2017). For example, Pepper and Nettle (2014a) found that experimentally priming the perceived controllability of mortality risk influences health-related decision making. They found that when participants were primed to perceive mortality as an uncontrollable (extrinsic) risk, they were more likely to choose an unhealthy food reward in preference to a healthy alternative. Thus, it is important to understand the extent to which the risks of the pandemic are perceived as extrinsic, and to test for associations between perceived extrinsic risks and health behaviour.

In addition to affecting general health behaviours, perceptions of risk may also influence the extent to which people engage with infection prevention behaviours. Compliance with preventative measures designed to prevent the spread of disease has been suggested to be associated with the public’s perception of risk (Brug et al. 2009). Individual perceptions of personal vulnerability to a specific risk may also play a key role in the behavioural response to risk (Millstein and Halpern-Felsher 2002). For example, the first global examination of public risk perception with respect to COVID-19 found that perceptions of COVID-19 related risk were significantly correlated with the reported adoption of preventative measures (including washing hands, wearing masks, and physical distancing) in all ten countries included in the sample (total sample *n* = 6991; Dryhurst et al. 2020).

There have been urgent calls for research into the psychological factors involved in the public response to COVID-19 (Asmundson and Taylor 2020). The spread of disease is affected by individual behaviour, which in turn is influenced by perceptions of risk (Ibuka et al. 2010). The pervasiveness of media coverage has also been shown to exacerbate the severity of perceived risk (Young et al. 2013). Furthermore, new risks are more likely to be perceived as uncontrollable (de Zwart et al. 2010). We predict that, due to the novelty of COVID-19 and the extensive media coverage, many people may perceive it as being a mortality risk beyond their control, which may have downstream behavioural consequences. More information is needed to understand the relationships between perceptions of risk and health behaviours during the outbreak of COVID-19 (Betsch et al. 2020). To address this, we have examined how risk perceptions were associated with self-reported behaviour during the strictest period of the first lockdown in the UK. Based on our findings, we make suggestions towards improving the effectiveness of public health strategies in the future.

## Method

This study was approved by the Department of Psychology Ethics Committee (23857) at Northumbria University. Our measures, predictions, and analytical plan are registered with the Open Science Framework [https://osf.io/8jqsn/].

For our study, 514 adults were anonymously surveyed using a Qualtrics questionnaire delivered by the platform Prolific [www.prolific.co], a company that offers a high-quality participant pool of research-participant volunteers, and provided a nationally-representative sample of UK participants. To provide a nationally representative sample, Prolific screens participants based on age, gender, and ethnicity in proportion to data derived from the UK’s latest national census (Office for National Statistics 2013; Prolific Team 2019). Although no sample can be fully representative of a population across all measures (Zhang et al. 2017), Prolific’s screening method has been validated as an effective stratified sampling tool for providing nationally representative samples during the COVID-19 pandemic (Kooistra et al. 2020). The target sample size of 500 was based on suggested guidelines for conducting surveys in exploratory research (Daniel 2012).

The survey was launched on 6 May 2020, and closed on 7 May 2020. For context, the largest number of registered deaths in England and Wales occurred during the week ending 17 April 2020 (Office for National Statistics 2020a, b). However, the UK became the first country in Europe to surpass 30,000 COVID-19 related deaths on 6 May 2020, the day our survey was launched, meaning that the death rate would have been salient in the media at the time (UK Government 2020). Thus, our findings report the perceptions and behaviours of participants after the initial peak of the pandemic, but still within the strictest period of the first UK lockdown (Cabinet Office 2020a).

We excluded 16 participants from our analysis due to inconsistent survey responses for age and gender on our survey, when compared to the responses on their Prolific profile. Two further participants were excluded as extreme outliers, having reported knowing 200 or more people who had contracted COVID-19. Participants were asked their age, gender, ethnicity, and National Statistics Socio-economic Classification (NS-SEC). Our final sample comprised 496 participants: 254 females and 242 males, aged 19–85 (mean age = 45.95, SD = 15.41). The questionnaire is available as part of our pre-registration on the Open Science Framework [https://osf.io/8jqsn/]. In the same survey, data were also collected on information-seeking behaviours and experiences of COVID-19. These findings are reported in “Information seeking, personal experiences, and their association with COVID-19 risk perceptions: demographic and occupational inequalities” (Brown et al. 2020, 2021).

### Perceptions of risk

Participants provided a measure of perceived extrinsic mortality risk by stating a score for their believed likelihood of living to 81 (the current average UK life expectancy), provided they make the maximum effort to look after their health (on a scale from 0, no chance, to 100, certain). The score was then subtracted from 100: Perceived extrinsic mortality risk is the difference between 100% certainty of living to 81 and the perceived likelihood of living to 81 with maximum health effort (Pepper and Nettle 2014b). This reflects the ‘extrinsic’ portion of mortality risk, or the portion of risk which the participant believes is beyond their control. Two perceived extrinsic mortality risk scores were recorded. Firstly, a score that takes the effects of the current pandemic into consideration. Secondly, an estimated score for how participants felt they would have responded without the effects of the current pandemic. The difference between these scores was used to determine the influence of the pandemic on perceived extrinsic mortality risk.

Participants also provided a measure of perceived risk of infection by stating a score for their believed likelihood of contracting COVID-19, provided they made the maximum effort to follow what were Government-recommended preventative measures at the time (see below, section ‘Adherence to preventative measures’). This was reported, again on a scale from 0 (no chance) to 100 (certain) of being infected. A score for perceived threat to life from COVID-19 was also recorded, again with a scale ranging from 0 (not at all a threat to life), to 100 (absolutely a threat to life). Finally, participants rated both their concern about and perceived degree of control over preventing the spread of COVID-19 to others, in the event that they become infected. All scores for perceptions of risk were on a scale from 0 to 100. For our analysis of perceptions of risk, we excluded 19 participants who reported having been infected with COVID-19. This was because having personally had COVID-19 would be likely to tilt their responses with regard to perceived risk of infection towards certainty, and their responses with regard to perceived threat to life towards zero.

### Adherence to preventative measures

Participants were asked about the degree to which they were adhering to measures designed to prevent the spread of infection during the outbreak of COVID-19. They indicated their adherence by selecting answers on a seven-point Likert scale for how often they were following specific measures, ranging from ‘never’ to ‘always’. The questions asked were about adherence to the following six preventative measures, which were recommended by the UK Government and the NHS at the time of conducting the survey:

1. “Only go outside of your home for food, health reasons or work (but only if you cannot work from home).”
2. “If you do go outside of your home, stay 2 metres (6ft) away from other people at all times.”
3. “Do not go outside of your home to meet others, even friends or family.”
4. “Wash your hands with soap and water often, making sure to do this for at least 20 seconds.”
5. “Cover your mouth and nose with a tissue or your sleeve (not your hands) when you cough or sneeze.”
6. “Do not touch your eyes, nose or mouth if your hands are not clean.”

### General health behaviours

Participants were asked to indicate the degree to which they were adhering to general public health advice recommended by the NHS at the time of the survey. Participants indicated their adherence by selecting answers on a seven-point Likert scale for how often they were following specific recommendations, ranging from ‘never’ to ‘always’. The questions asked were about adherence to the following health advice:

1. “Eat at least 5 portions of a variety of fruit and vegetables every day.”
2. “Avoid regularly drinking more than 14 units of alcohol per week.”
  (14 units is equivalent to a bottle and a half of wine or five pints of export-type lager (5% abv) over the course of a week — this applies to both men and women)
3. “Do at least 150 minutes of moderate intensity activity a week or 75 minutes of vigorous intensity activity a week.”
  (One way to tell if you are working at a moderate intensity level is if you can still talk, but not sing. Vigorous intensity activity makes you breathe hard and fast. If you are working at this level, you will not be able to say more than a few words without pausing for breath)

Participants also answered the question “do you smoke” by selecting an answer on a seven-point Likert scale, ranging from ‘never’ to ‘always’. This measure was reverse-scored, so a higher score reflects the degree to which participants were adhering to general public health advice not to smoke.

### Analysis

All statistical analyses were performed using R (R Core Team 2019). The R script used for data processing and analysis is available alongside our preregistration. The following packages were used for data processing, analysis, and data visualisation: tidyverse (Wickham 2017), tidyr (Wickham and Henry 2019), pysch (Revelle 2018), MASS (Venables and Ripley 2002), and apaTables (Stanley 2018).

Our main variables are categorised under four key themes: 1) demographics, 2) risk perceptions, 3) general health behaviours, and 4) COVID-19 prevention behaviours. For each regression analysis presented, we first ran analyses to look for any demographic differences in perceptions and behaviours. Our demographic predictors included age, gender, and NS-SEC. Any significant demographic predictors were then included as control variables in subsequent models. Since compliance with health advice was measured on a seven-point Likert scale, we ran a series of ordinal logistic regression models to assess whether each of the reported behaviours was predicted by perceptions of risk. Continuous predictors in the ordinal models were standardised to aid the comparison of odds ratios. Paired-samples *t*-tests were used to assess the difference in perceived extrinsic mortality risk with and without taking the risks of the pandemic into account, and the difference between our measures of perceived control over catching COVID-19 and perceived control over spreading it.

## Results

### Descriptive statistics

Table 1 presents the descriptive statistics for our sample, whose ages ranged from 19 to 85 (M = 45.95, SD = 15.41).

**Table 1:** Sample characteristics for age, gender, ethnicity, and occupational class

	Category	Number (*n* = 496)	Percentage of sample
Age	18–34	137	27.62
	35–49	140	28.23
	50–64	160	32.26
	65+	59	11.90
Gender	Female	254	51.21
	Male	242	48.79
Ethnicity	White	400	80.65
	Asian	42	8.47
	Black	24	4.84
	Mixed	16	3.23
	Other	14	2.82
Occupational class (NS-SEC) (*n* = 393)	1.1 Large employers and higher managerial and administrative occupations	11	2.80
	1.2 Higher professional occupations	58	14.76
	2 Lower managerial, administrative and professional occupations	74	18.83
	3 Intermediate occupations	75	19.08
	4 Small employers and own account workers	13	3.31
	5 Lower supervisory and technical occupations	8	2.04
	6 Semi-routine occupations	32	8.14
	7. Routine occupations	25	6.36
	8 Never worked and long-term unemployed	97	25.68

### Perceptions of risk

A paired *t*-test showed a significant difference of 4.68% on average between perceived extrinsic mortality risk scores that took the effects of the pandemic into consideration (M = 32.73) and those that estimated the level of perceived risk that would have been experienced without the effects of the pandemic (M = 28.06, *t*(495) = 8.60, *p* < .001) (see supplement, Tables S1–2, for descriptive and correlational statistics for all measures of perceived COVID-19 related risk). Overall, 54% of our sample reported a difference in perceived extrinsic mortality risk when taking the effects of the pandemic into account. For one third of our sample, there was no difference in perceived risk when taking the effects of the pandemic into consideration compared with not doing so. Just over a third reported an increase of between 1 and 10%, one fifth reported an increase of over 20%, and the remainder of the sample reported a reduction in perceived risk when taking the effects of the pandemic into consideration (see supplement, Table S3).

Participants felt more able to control whether they would contract COVID-19 themselves (M = 74.12%) than whether they would spread the infection to others in the event that they became infected (M = 63.44%, *t*(495) = 7.05, *p* < .001).

We predicted that perceived extrinsic mortality risk, accounting for the pandemic, would be affected by a combination of perceived risk of infection and perceived threat to life. Perceived threat to life was predictive of the difference between perceived extrinsic mortality risk scores that took the outbreak of COVID-19 into consideration and scores that did not; *b* = .07, (95% CI = .02, .13), * p* < .01. However, perceived risk of infection was not predictive of this difference (see supplement, Table S4).

With respect to our demographic predictors of COVID-19 related risk perceptions (see supplement, Tables S5–11), age was found to predict higher levels of perceived threat to life (Table S6). Being male predicted lower levels of perceived threat to life (Table S6), as well as higher levels of perceived extrinsic mortality (when considered separate to the effects of the pandemic (Table S8). Being male also predicted being less concerned about spreading the virus to others in the event of personal infection (Table S10). Simplified NS-SEC was not associated with any of our measures of risk perception (Tables S5–11). Significant demographic predictors were included as control variables in all subsequent regression models pertaining to perceptions of risk.

### General health behaviour during the pandemic

**Fig. 1:**
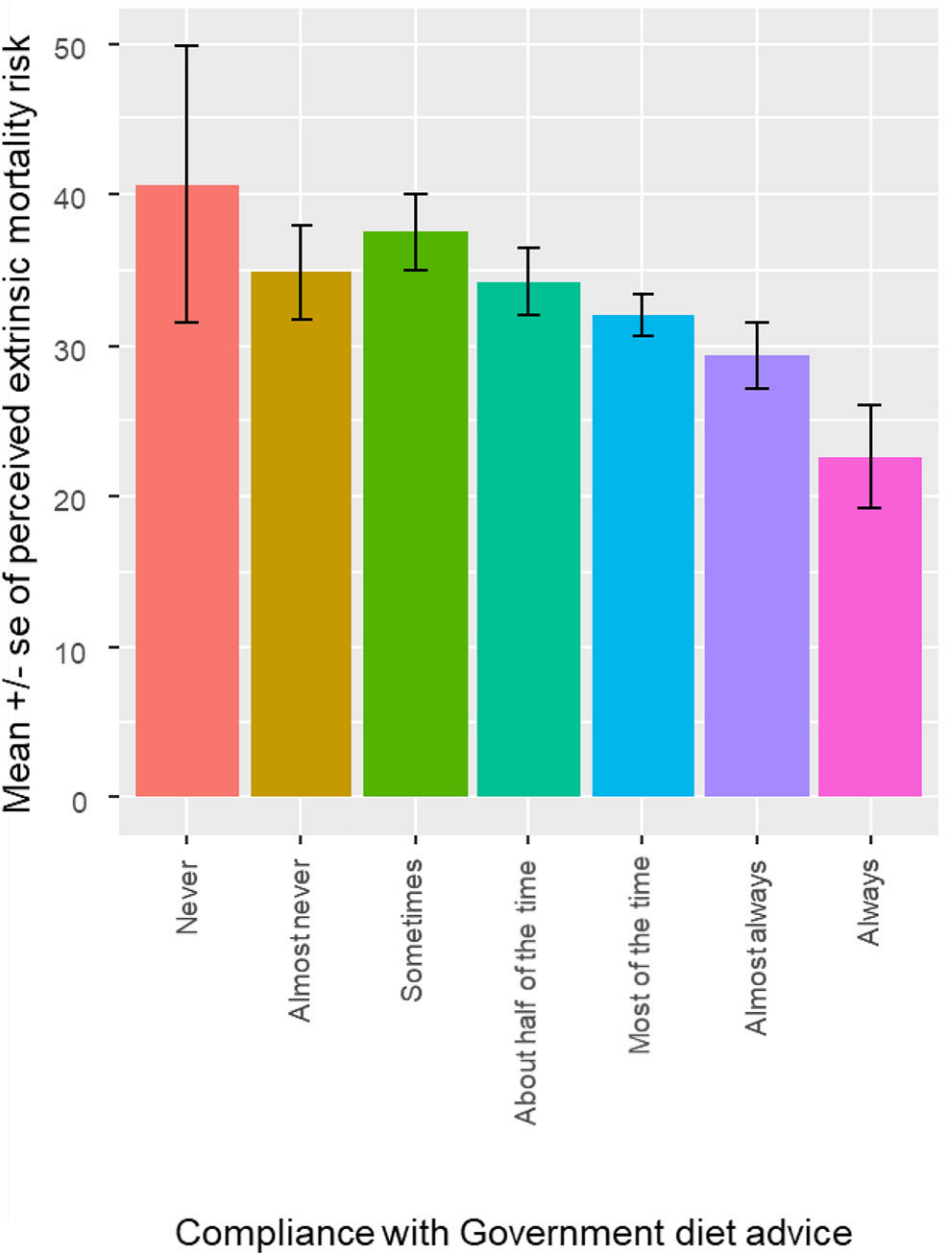
Association between perceived extrinsic mortality risk, taking the pandemic into account, and reported adherence to dietary recommendations (total sample minus those personally infected with COVID-19, *n* = 477)

**Fig. 2:**
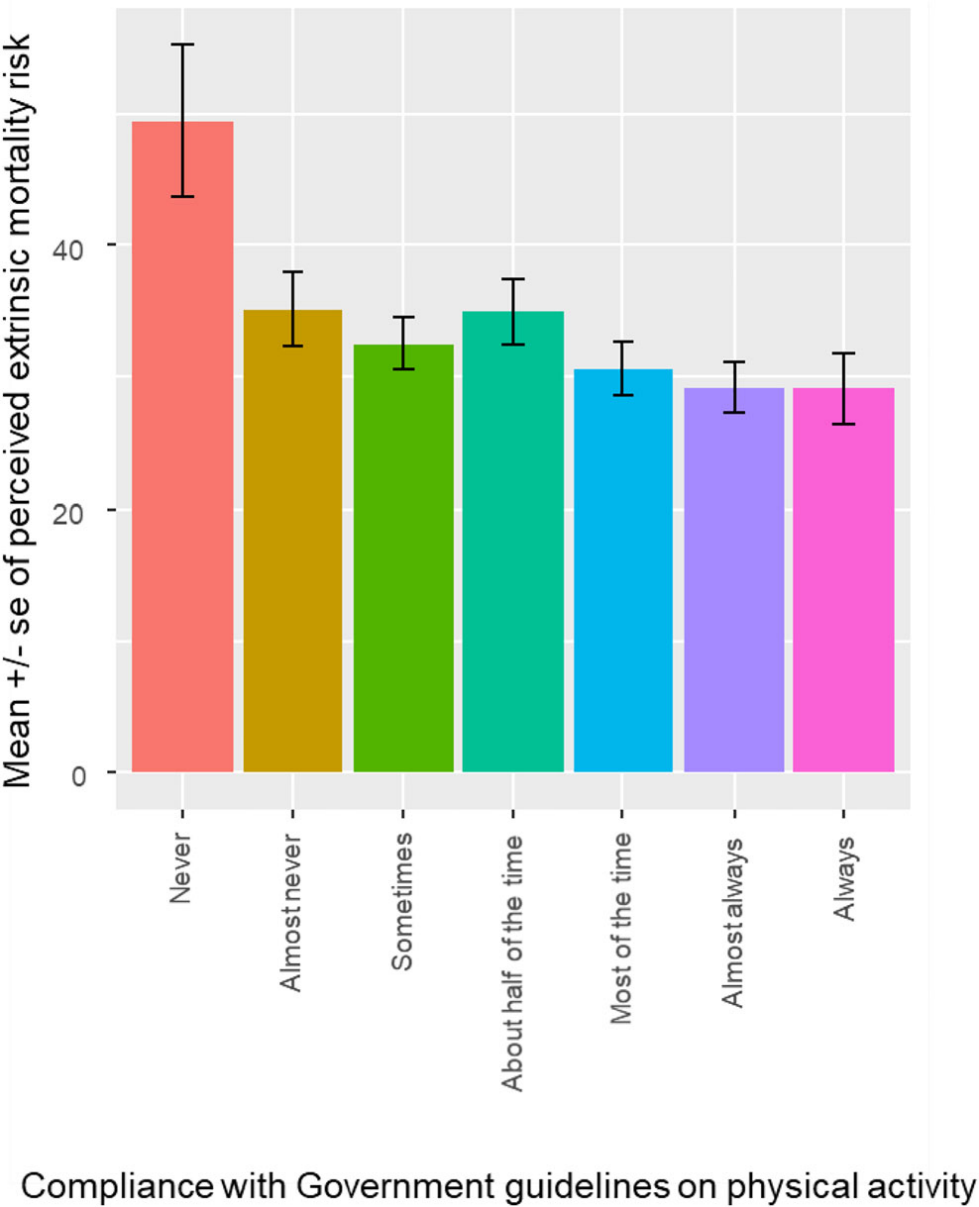
Association between perceived extrinsic mortality risk, taking the pandemic into account, and adherence to physical activity guidelines (total sample minus those personally infected with COVID-19, n = 477)

**Fig. 3:**
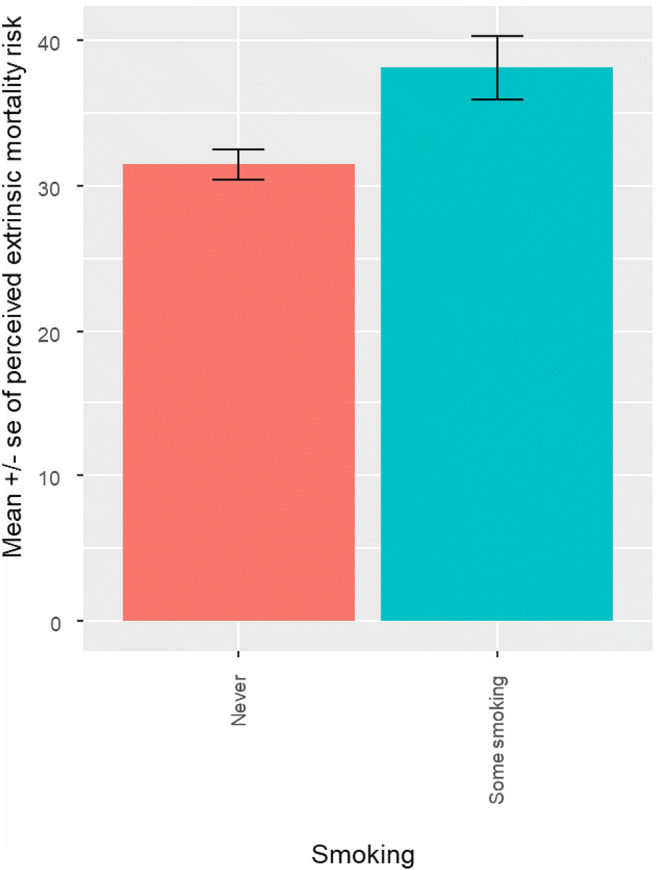
Association between perceived extrinsic mortality risk, taking the pandemic into account, and frequency of smoking (total sample minus those personally infected with COVID-19, *n* = 477)

Greater perceived extrinsic mortality risk when taking the pandemic into account was associated with lower adherence to dietary advice (β = −.29, s.e. = .08, OR = 0.75, 95% CIs = 0.63, 0.88; see Fig. 1). Controlling for the known effect of gender (β = 0.40, s.e. = 0.16, OR = 1.49, 95% CIs = 1.09, 2.05), perceived extrinsic mortality risk was also associated with lower reported adherence to physical activity guidelines (β = −.32, s.e. = .09, OR = 0.72, 95% CIs = 0.61, 0.86; see Fig. 2), and with greater reported frequency of smoking (β = −0.30, s.e. = 0.11, OR = 0.74, 95% CIs = 0. 59, 0.93; see Fig. 3), even when controlling for the effect of socioeconomic status (NS-SEC, β = − 0.26, s.e. = 0.12, OR = 0.77, 95% CIs = 0.60, 0.98).

Perceived threat to life was also associated with lower adherence to physical activity guidelines (β = −.18, s.e. = .09, OR = 0.83, 95% CIs = 0.70, 1.00).

For an overview of the frequencies for the different reported levels of compliance with the UK Government’s recommendations regarding diet, alcohol consumption, physical activity, and smoking during the outbreak of COVID-19, see the supplement (Fig. S1 and Table S12).

### Adherence to preventative measures

The median reported adherence to government measures designed to prevent the spread of COVID-19 infection was “almost always”, with the exception of avoiding touching one’s eyes, nose, or mouth with unclean hands, which, on average, participants only reported adhering to “most of the time” (see supplement, Fig. S2). 74.4% of our sample reported always adhering to advice not to meet others outside of the home. Similarly, 65.12% reported always adhering to advice to stay at home. However, only 23.59% reported always adhering to advice to not touch one’s face with unclean hands (see supplement, Table S13).

Our demographic predictors did not predict adherence to COVID-19 advice to stay at home, stay 2 m from others when out of the home, or avoid meeting others. However, being male was predictive of lower levels of adherence to preventative hygiene measures: handwashing (β = −.69, s.e. = .19, OR = 0.50, 95% CIs = 0.34, 0.73), covering one’s mouth when coughing (β = −.60, s.e. = .20, OR = 0.55, 95% CIs = 0.37, 0.81) and not touching one’s face with unclean hands (β = −.89, s.e. = .19, OR = 0.41, 95% CIs = 0.28, 0.59).

Perceived threat to life was positively associated with adherence to five out of six preventative measures (the exception being not meeting others outside of the home) and concern about spreading infection to others was associated with four out of six preventative measures (the exceptions being keeping a 2 m distance from others and not touching one’s face; see Table 2).

**Table 2:** Results from ordinal logistic regression analyses showing predictors of adherence to infection prevention measures

Outcome	Stay home		Keep 2 m distance		Do not meet others		Wash hands 20+ seconds		Cover mouth when coughing		Do not touch eyes/nose/mouth	
	Β (s.e.)	OR (CIs)	Β (s.e.)	OR (CIs)	Β (s.e.)	OR (CIs)	Β (s.e.)	OR (CIs)	Β (s.e.)	OR (CIs)	Β (s.e.)	OR (CIs)
Predictor												
Perceived threat to life	0.39* (0.11)	1.48* (1.20, 1.83)	0.40* (0.10)	1.49* (1.23, 1.80)	0.15 (0.11)	1.16 (0.93, 1.46)	0.45* (0.09)	1.57* (1.31, 1.90)	0.25* (0.10)	1.29* (1.06, 1.56)	0.47* (0.09)	1.61* (1.34, 1.92)
Concern about spreading infection	0.25* (0.09)	1.29* (1.08, 1.54)	0.18 (0.09)	1.20 (1.00, 1.43)	0.21* (0.10)	1.23* (1.01, 1.49)	0.21* (0.09)	1.24* (1.04, 1.47)	0.19* (0.09)	1.21* (1.02, 1.44)	−0.01 (0.08)	0.99 (0.84, 1.16)
Perceived risk of infection	−0.27* (0.09)	0.77* (0.64, 0.92)	−0.18* (0.09)	0.83* (0.70, 0.99)	−0.19 (0.10)	0.83 (0.68, 1.01)	−0.04 (0.09)	0.96 (0.81, 1.14)	0.01 (0.09)	1.01 (0.85, 1.21)	0.02 (0.08)	1.02 (0.87, 1.21)
Perceived control over spreading infection	0.16 (0.10)	1.29 (1.08, 1.54)	0.05 (0.09)	1.05 (0.88, 1.25)	0.07 (0.11)	1.08 (0.87, 1.32)	−0.00 (0.09)	1.00 (0.84, 1.18)	0.17 (0.09)	1.19 (1.00, 1.42)	0.27* (0.08)	1.31* (1.11, 1.54)
Perceived extrinsic mortality risk (with pandemic)	0.04 (0.10)	1.04 (0.86, 1.27)	−0.06 (0.09)	0.94 (0.77, 1.12)	0.02 (0.11)	1.02 (0.83, 1.27)	−0.17 (0.09)	0.85 (0.71, 1.00)	−0.07 (0.09)	0.94 (0.78, 1.11)	−0.12 (0.08)	0.86 (0.75, 1.04)
Sex (male)	NA		NA		NA		−0.69* (0.19)	0.50* (0.34, 0.73)	−0.60* (0.20)	0.55* (0.37, 0.81)	−0.89* (0.19)	0.41* (0.28, 0.59)

Each outcome was modelled separately. *OR* = Odds ratio, *CI* = 95% Confidence interval, *statistically significant (t > 2), NA = not applicable because sex was only included as a control variable, where it was a significant predictor of risk perception in prior demographic models

## Discussion

This study measured perceptions of COVID-19 related risk, and explored the impact of these on both general health behaviours and adherence to measures designed to prevent the spread of infection. Our findings reflect the experience of participants after the initial peak of the pandemic, but still within the strictest period of the first UK lockdown (Cabinet Office 2020a). As predicted, we found that perceptions of risk were associated with both general health behaviours and levels of adherence to COVID-19 prevention measures.

### Perceptions of risk

Perceived extrinsic mortality risk scores that took the effects of the pandemic into consideration were, on average, 5% higher than those that did not. The extent to which COVID-19 is perceived as an extrinsic mortality risk varied across our sample; however, the average response was a small increase in perceived risk when taking the pandemic into consideration. The Uncontrollable Mortality Risk Hypothesis predicts that people with increased perceived extrinsic mortality risk are likely to be less motivated to engage in positive health behaviours (Pepper and Nettle 2014a), and this prediction was supported by our data. Though we lack longitudinal data to allow us to assess the true extent to which the pandemic has affected health behaviour, our results suggest that the small increase in perceived extrinsic mortality risk which was generated by the pandemic may have disincentivised health behaviours. We found that perceived threat to life, but not perceived risk of infection, was predictive of this pandemic-related increase in perceived extrinsic mortality risk.

### General health behaviour

On average, our sample reported “almost always” adhering to health advice concerning alcohol consumption during the pandemic, following dietary advice “most of the time” and meeting recommended levels of physical activity “about half of the time”. Furthermore, 81% of our sample reported that they never smoked.

Greater perceived extrinsic mortality risk was associated with lower levels of adherence to dietary advice and to recommended levels of physical activity. Higher perceived extrinsic mortality risk was also associated with lower incidence of not smoking. This provides additional support for the Uncontrollable Mortality Risk Hypothesis, which predicts that people who believe they are more likely to die due to factors beyond their control should be less motivated to engage in positive health behaviours (Pepper and Nettle 2014a, b). Although we do not have the longitudinal data needed to demonstrate changes in behaviour as a result of the pandemic, this result suggests that those who are experiencing higher levels of perceived extrinsic mortality risk during the pandemic may be less likely to engage in positive health behaviours, such as a good diet and physical activity. This is worrying, given that an unhealthy diet may lead to worse health outcomes regarding the susceptibility to, recovery from, and long-term effects of COVID-19 (Butler and Barrientos 2020). Lower levels of physical activity during the pandemic may also decrease the ability to resist viral infection and contribute towards the risk of long-term negative health outcomes (Woods et al. 2020). This suggests that those who are experiencing greater perceived extrinsic mortality risk during the pandemic may be more likely to respond in a way which puts them at greater risk in the event that they become infected with COVID-19. The UK Government has recognised the possibility that COVID-19 will continue to circulate in society on a long-term basis (Cabinet Office 2020b). Therefore, it is possible that the effects on perceived extrinsic mortality risk, and associated health behaviours may not be limited to the current pandemic, but could endure over time to reflect the ongoing threat of COVID-19.

Perceived threat to life was also predictive of lower adherence to recommended levels of physical activity. We speculate that this may be because those who consider COVID-19 to pose a greater threat to life are less likely to leave their home to exercise, due to potential exposure to others and increased risk of infection. It is noted that Government recommendations in response to COVID-19 were focused on social distancing measures (Cabinet Office 2020a) and did not provide specific health guidance with regard to diet, exercise, smoking, and alcohol consumption. Our study measured self-reported adherence to general health advice available from the UK Government and NHS at the time of the study. Given the discussed associations between general health behaviours and COVID-19 health outcomes, it is possible that the absence of advice about maintaining general health and fitness during lockdown may have impacted on the susceptibility to negative health outcomes of those infected with COVID-19 (Butler and Barrientos 2020; Woods et al. 2020).

### Adherence to preventative measures

On average, participants reported “almost always” adhering to government measures designed to prevent the spread of COVID-19 infection, with the exception of avoiding touching one’s eyes, nose or mouth, which, on average, participants reported adhering to “most of the time”. This suggests a reasonably high level of overall compliance with the Government’s earlier recommendations in response to the pandemic. However, there were notable differences in degrees of reported compliance, most apparent when comparing genders. Being male was predictive of lower levels of adherence to hygiene measures recommended by the NHS. This finding is consistent with research into gender differences in compliance with measures designed to prevent the spread of infection, in which male healthcare workers are less compliant than their female counterparts (Ward 2004). A variety of biological, social, and occupational explanations have been suggested for explaining gender differences in infection control (Ward 2004); however, a potential mechanism is provided by the construct of disgust. Disgust is thought to have evolved as a disease-avoidance mechanism for protecting us against contracting infectious disease (Oaten et al. 2009). In response to the threat of infection, disgust is associated with promoting hygiene behaviour (Curtis et al. 2011) and men have consistently been found to have lower levels of disgust than women (Skolnick 2013). Al-Shawaf et al. (2017) put forward various hypotheses for why women may have evolved higher levels of disgust towards pathogens than men, including to avoid transmitting infections to their offspring. They also suggest that lower levels of disgust in males may serve an evolutionary benefit in signalling a strong immune system to facilitate mating, as well as potential benefits for both hunting and warfare. Men may therefore report lower levels of adherence to hygiene measures designed to prevent the spread of infection because they typically experience lower levels of disgust than women.

A range of risk perception variables were predictive of levels of compliance to preventative measures. This provides support for the notion that compliance with disease prevention measures is associated with the public’s perception of risk (Brug et al. 2009). Research during the current pandemic has also found that risk perception is positively correlated with adherence to a variety of preventative measures related to social distancing and hygiene (Dryhurst et al. 2020). The most notable predictor of adherence from our sample was perceived threat to life from COVID-19, which was positively associated with higher levels of compliance with five of the six preventative measures. This provides some support for the findings of early research into the response to the pandemic in the UK, which found that the sole predictor of public health compliance was fear of COVID-19 (Harper et al. 2020). Harper et al. (2020) argued that fear may induce a functional response to the pandemic through increased compliance with health measures. However, given that fear appeals may also increase perceived extrinsic mortality risk, potentially thereby decreasing other health-promoting behaviours, we would recommend focusing on approaches that make the threat appear more controllable. Indeed, others have suggested that fear communications are more effective when people believe that they have the capacity to respond to the threat (Peters et al. 2018). A recent meta-analysis of the utility of fear appeals found that their effectiveness increases when accompanied by statements of efficacy (Tannenbaum et al. 2015). Statements of efficacy provide information regarding an individual’s ability to effectively respond to a threat, as well as promoting the utility of the proposed response (Mongeau 2020). In the context of the current pandemic, statements of efficacy may emphasise the utility of proposed COVID-19 prevention measures, as well as highlighting an individual’s ability to protect themselves from infection by complying with these measures. Current research into compliance with COVID-19 prevention measures in response to the pandemic has found that feelings of efficacy are effective in motivating compliance (Jørgensen et al. 2020). Given the importance of including statements of efficacy in health communications, future research should look to evaluate the effectiveness of specific health messages during the pandemic to better understand how they can be utilised in future public health strategies.

The second most consistent predictor of adherence to preventative measures from our risk perception variables was concern over spreading the infection to others. This measure was associated with four out of six of the infection control measures suggesting that, in addition to threat to life, individuals are also motivated to comply with public health strategies by their concern for others. This motivation may be especially pertinent to compliance with additional preventative behaviours that are more relevant to preventing the spread of infection than personally avoiding infection, such as mask wearing. Compliance with such measures may rely on a shift in focus from self-protection to more altruistic behaviour (Cheng et al. 2020).

### Limitations

The results of this study are not without limitation. Firstly, we emphasise that all of the behavioural measures are self-reported. It is possible that these self-reported measures have been affected by participant response biases to reflect social norms with regard to compliance with public health measures during the pandemic. Further studies may seek to incorporate objective measures of adherence to recommended behaviours.

Additionally, we recognise that during the outbreak of a new viral threat, the public’s perceptions of risk and associated behaviours are likely to evolve in response to constantly changing information and policies throughout the course of the outbreak. The data from our sample were captured at a single point in time during the initial lockdown, therefore our findings will not reflect any ongoing changes in perception and behaviour as the pandemic progresses. Further research may collect data at several time points to reflect how perceptions and behaviours vary over time.

Finally, since we do not have longitudinal data, we cannot be certain that the perceived extrinsic mortality risk generated by the pandemic has affected health behaviours. We can only establish that 1) perceived extrinsic mortality risk was associated with poorer self-reported compliance with recommended general health behaviours, and 2) that, on average, participants reported greater perceived extrinsic mortality risk when considering the risk of COVID-19 than when they were asked to discount the risks resulting from the pandemic.

## Conclusion

Our most consistent predictor of compliance with COVID-19 prevention measures, was perceived threat to life. Elevated levels of perceived threat may therefore increase compliance with measures designed to prevent the spread of infection. However, we also found that perceived threat to life was associated with a reduction in physical activity, and was a predictor of increased perceived extrinsic mortality risk, which was broadly associated with lower engagement with health-promoting behaviours. From a public health perspective, this suggests that promoting a message that highlights threat to life may be effective in raising levels of adherence to measures of infection control but may ultimately lead to a reduction in positive health behaviours, potentially jeopardising the ability of some individuals to effectively respond to viral infection. This conclusion supports previous research into appealing to fear in public health messaging which found that fear and perceived threats to life can produce a complex set of reactions which include both adaptive and maladaptive health behaviours (Arndt et al. 2006). We suggest that fear communications should be accompanied by statements of efficacy so that the recipients feel more able to control the threat. Concern over spreading infection to others was our second most consistent predictor of compliance. Due to the complex range of behavioural outcomes that feelings of threat to life may induce, public health strategies that seek to evoke feelings of concern for others may be better for promoting compliance with anti-infection measures whilst avoiding unintended consequences.

## Supplementary Information

ESM 1 (PDF 528 kb)

## Data Availability

In the spirit of full transparency, we agree to make the analysis scripts and data used for the analysis contained within this submission available via the Center for Open Science (osf.io) or via an alternative means upon request by the publisher.
