# Supplementary material for: COVID-19: the relationship between perceptions of risk and behaviours during lockdown
Source: Z Gesundh Wiss. 2021 May 13;31(4):623–33. doi: 10.1007/s10389-021-01543-9 (PMC8118375; doi:10.1007/s10389-021-01543-9)
Supplement: Supplementary file 1 — (PDF 528 kb) [file 10389_2021_1543_MOESM1_ESM.pdf]

Supplement

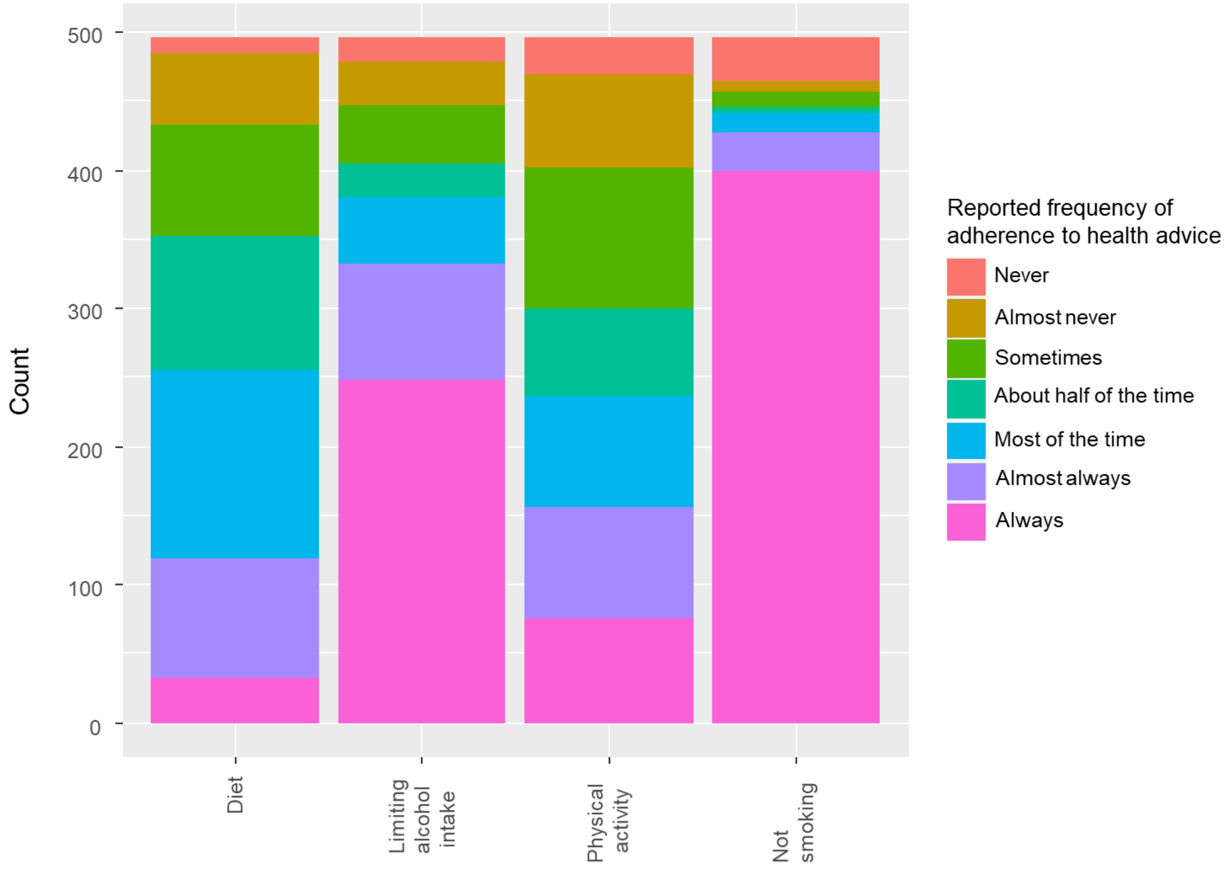

Figure S1. Frequency of reported compliance with UK Government health recommendations (total sample, n = 496).

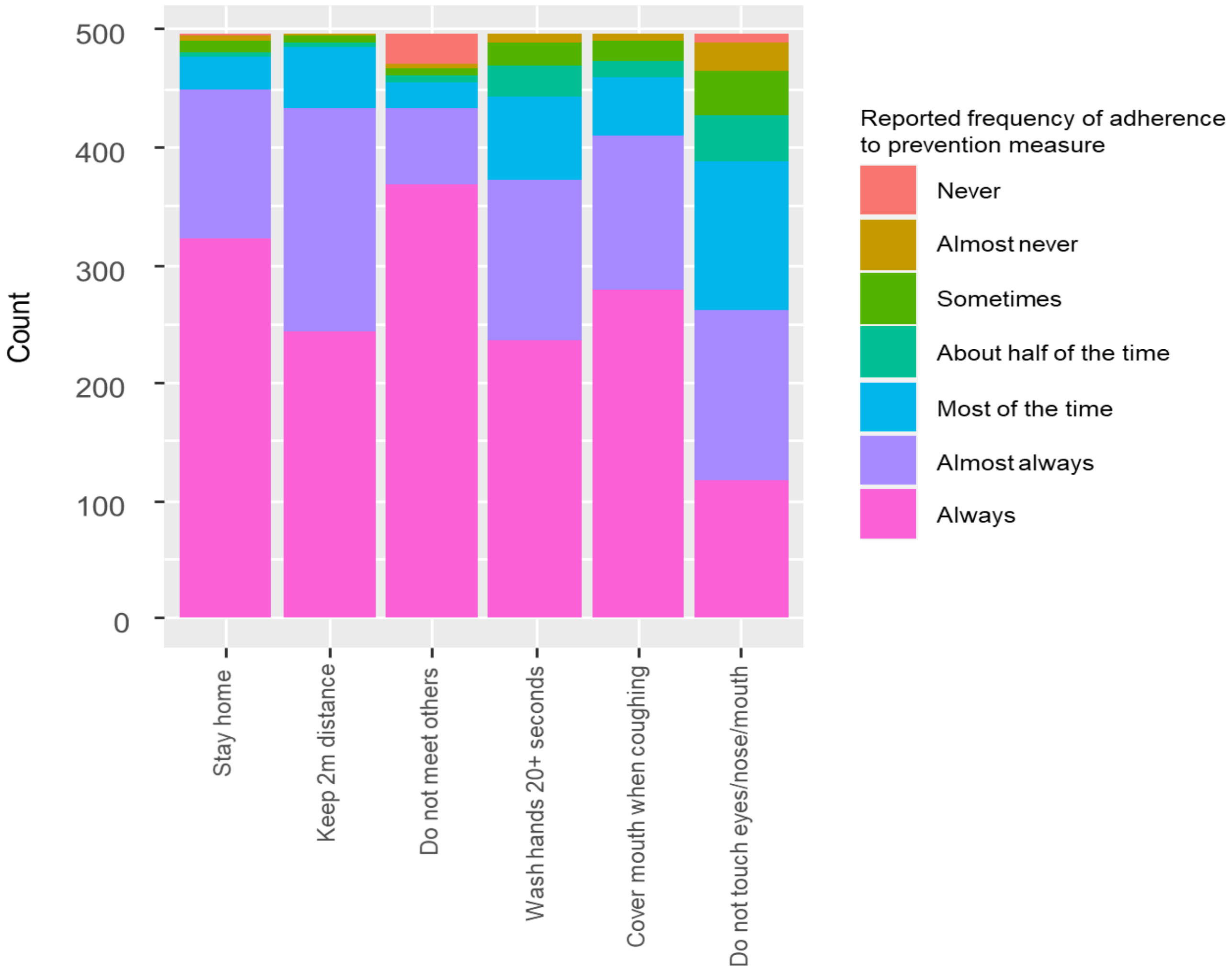

Figure S2. Frequency of adherence to UK Government preventative measures for COVID-19 (total sample, n = 496).

Table S1. Descriptive statistics for perceptions of COVID-19 related risk

	Mean	Median	SD	Min	Max
**Perceived risk of infection**	25.89	20	21.55	0	100
**Perceived threat to life**	46.39	49	31.80	0	100
**Perceived extrinsic mortality risk with the pandemic**	32.73	29	21.07	0	100
**Perceived extrinsic mortality risk without the pandemic**	28.06	21	20.93	0	100
**Perceived extrinsic mortality risk difference**	4.68	1	12.12	-80	69
**Concern over spreading the infection to others**	83.51	92	23.17	0	100
**Perceived control over spreading infection to others**	63.44	70	26.17	0	100

Number = 477 for all variables. SD = Standard deviation, Min = Minimum, Max = Maximum.

Table S2. Means, standard deviations, and correlations for risk perception variables

Variable	*M*	*SD*	1	2	3	4	5	6
1. Perceived risk of COVID-19 infection	25.89	21.55						
2. Perceived threat to life	46.39	31.80	.18**					
3. Perceived extrinsic mortality risk with the pandemic	32.73	21.07	.02	.18**				
4. Perceived extrinsic mortality risk without the pandemic	28.06	20.93	.04	.09*	.83**			
5. Perceived extrinsic mortality risk difference	4.68	12.12	-.03	.16**	.30**	-.28**		
6. Concern over spreading the infection to others	83.51	23.17	.04	.29**	-.03	-.06	.04	
7. Perceived control over spreading infection to others	63.44	26.17	-.01	-.12**	-.15**	-.14**	-.01	.04

*Note. M and SD are used to represent mean and standard deviation, respectively. * indicates p < .05. ** indicates p < .01. n = 477 for all variables.*

Table S3. Frequency and percentage of the difference between scores of perceived extrinsic mortality risk that took the 2
effects of the pandemic into consideration compared to those that did not.

Difference between scores	Frequency	Percentage of sample
Decrease in score	63	12.7
No change	165	33.3
1-10% increase	168	33.9
11-20% increase	61	12.3
21-30% increase	27	5.4
31% + increase	12	2.4

n = 477

Table S4. Regression results examining how perceived risk of contracting COVID-19 despite following Government recommendations, and perceived threat to life from COVID-19 predict the portion of perceived extrinsic mortality risk that is due to the pandemic

Predictor	*b*	*b* 95% CI [LL, UL]	sr^2	sr^2 95% CI [LL, UL]	Fit
(Intercept)	2.28	[-0.51, 5.07]			
Perceived risk of COVID-19 infection	-0.02	[-0.12, 0.07]	.00	[-.00, .00]	
Perceived threat to life	0.07**	[0.02, 0.13]	.02	[-.01, .04]	
Perceived risk of COVID-19 infection: Perceived threat to life	-0.00	[-0.00, 0.00]	.00	[-.00, .01]	
					R^2 = .030** 95% CI[.00,.06]

Note. *A significant b-weight indicates the semi-partial correlation is also significant. b represents unstandardized regression weights. sr2 represents the semi-partial correlation squared. LL and UL indicate the lower and upper limits of a confidence interval, respectively.*
** indicates p < .05. ** indicates p < .01.*

Table S5. Regression results assessing how age, gender, and simplified NS-SEC predict people's perceived risk of contracting COVID-19 despite following Government recommendations

Predictor	*b*	*b* 95% CI [LL, UL]	sr^2	sr^2 95% CI [LL, UL]	Fit
(Intercept)	28.67**	[19.58, 37.76]			
Age	-0.01	[-0.18, 0.16]	.00	[-.00, .00]	
Gender (Male)	-1.20	[-5.69, 3.29]	.00	[-.00, .01]	
Simplified NS-SEC	-0.26	[-1.11, 0.59]	.00	[-.01, .01]	
					R^2 = .002 95% CI[.00,.01]

Note. *A significant b-weight indicates the semi-partial correlation is also significant. b represents unstandardized regression weights. sr2 represents the semi-partial correlation squared. LL and UL indicate the lower and upper limits of a confidence interval, respectively.*
** indicates p < .05. ** indicates p < .01.*

Table S6. Regression results assessing how age, gender, and simplified NS-SEC predict people's perceived threat to life from COVID-19

Predictor	*b*	*b* 95% CI [LL, UL]	sr^2	sr^2 95% CI [LL, UL]	Fit
(Intercept)	34.01**	[21.30, 46.72]			
Age	0.24*	[0.01, 0.48]	.01	[-.01, .03]	
Gender (Male)	-10.60**	[-16.87, -4.32]	.03	[-.00, .06]	
Simplified NS-SEC	1.03	[-0.16, 2.22]	.01	[-.01, .02]	
					R^2 = .048** 95% CI[.01,.09]

Note. *A significant b-weight indicates the semi-partial correlation is also significant. b represents unstandardized regression weights. sr2 represents the semi-partial correlation squared. LL and UL indicate the lower and upper limits of a confidence interval, respectively.*
** indicates p < .05. ** indicates p < .01.*

Table S7. Regression results assessing how age, gender, and simplified NS-SEC predict people's perceived extrinsic mortality risk with the pandemic

Predictor	*b*	*b* 95% CI [LL, UL]	sr^2	sr^2 95% CI [LL, UL]	Fit
(Intercept)	25.31**	[16.29, 34.32]			
Age	0.07	[-0.10, 0.24]	.00	[-.01, .01]	
Gender (Male)	3.28	[-1.17, 7.73]	.01	[-.01, .02]	
Simplified NS-SEC	0.71	[-0.13, 1.55]	.01	[-.01, .02]	
					R^2 = .012<br>95% CI[.00,.04]

Note. *A significant b-weight indicates the semi-partial correlation is also significant. b represents unstandardized regression weights. sr2 represents the semi-partial correlation squared. LL and UL indicate the lower and upper limits of a confidence interval, respectively.*

** indicates p < .05. ** indicates p < .01.*

Table S8. Regression results assessing how age, gender, and simplified NS-SEC predict people's perceived extrinsic mortality risk when asked to think about what it would be without the pandemic

Predictor	*b*	*b* 95% CI [LL, UL]	sr^2	sr^2 95% CI [LL, UL]	Fit
(Intercept)	18.36**	[9.42, 27.31]			
Age	0.12	[-0.05, 0.28]	.00	[-.01, .02]	
Gender (Male)	5.62*	[1.20, 10.03]	.02	[-.01, .04]	
Simplified NS-SEC	0.66	[-0.18, 1.49]	.01	[-.01, .02]	
					R^2 = .024*<br>95% CI[.00,.06]

Note. *A significant b-weight indicates the semi-partial correlation is also significant. b represents unstandardized regression weights. sr2 represents the semi-partial correlation squared. LL and UL indicate the lower and upper limits of a confidence interval, respectively.*

** indicates p < .05. ** indicates p < .01.*

Table S9. Regression results assessing how age, gender, and simplified NS-SEC predict people's difference in perceived extrinsic mortality risk scores due to the pandemic

Predictor	*b*	*b* 95% CI [LL, UL]	sr^2	sr^2 95% CI [LL, UL]	Fit
(Intercept)	6.94**	[1.91, 11.97]			
Age	-0.05	[-0.14, 0.05]	.00	[-.01, .01]	
Gender (Male)	-2.34	[-4.82, 0.14]	.01	[-.01, .03]	
Simplified NS-SEC	0.05	[-0.42, 0.52]	.00	[-.00, .00]	
					R^2 = .012<br>95% CI[.00,.04]

Note. *A significant b-weight indicates the semi-partial correlation is also significant. b represents unstandardized regression weights. sr2 represents the semi-partial correlation squared. LL and UL indicate the lower and upper limits of a confidence interval, respectively.*

** indicates p < .05. ** indicates p < .01.*

Table S10. Regression results assessing how age, gender, and simplified NS-SEC predict people's degree of concern about spreading the virus in the event that they become infected

Predictor	*b*	*b* 95% CI [LL, UL]	sr^2	sr^2 95% CI [LL, UL]	Fit
(Intercept)	86.94**	[76.84, 97.04]			
Age	-0.06	[-0.25, 0.13]	.00	[-.01, .01]	
Gender (Male)	-5.46*	[-10.44, -0.47]	.01	[-.01, .03]	
Simplified NS-SEC	0.13	[-0.81, 1.08]	.00	[-.00, .00]	
					R^2 = .014<br>95% CI[.00,.04]

Note. A significant *b*-weight indicates the semi-partial correlation is also significant. *b* represents unstandardized regression weights. *sr2* represents the semi-partial correlation squared. *LL* and *UL* indicate the lower and upper limits of a confidence interval, respectively.
* indicates $p < .05$. ** indicates $p < .01$.

Table S11. Regression results assessing how age, gender, and simplified NS-SEC predict people's perceptions of control of spreading the virus in the event that they become infected

Predictor	*b*	*b* 95% CI [LL, UL]	sr^2	sr^2 95% CI [LL, UL]	Fit
(Intercept)	56.03**	[45.36, 66.71]			
Age	0.12	[-0.08, 0.32]	.00	[-.01, .02]	
Gender (Male)	0.77	[-4.50, 6.04]	.00	[-.00, .00]	
Simplified NS-SEC	0.22	[-0.78, 1.22]	.00	[-.00, .00]	
					R^2 = .004<br>95% CI[.00,.02]

Note. A significant *b*-weight indicates the semi-partial correlation is also significant. *b* represents unstandardized regression weights. *sr2* represents the semi-partial correlation squared. *LL* and *UL* indicate the lower and upper limits of a confidence interval, respectively.
* indicates $p < .05$. ** indicates $p < .01$.

Table S12. Frequency of adherence to UK Government health recommendations

Health Recommendation	Never	Almost never	Sometimes	About half of the time	Most of the time	Almost always	Always
Diet	11 (2.22%)	52 (10.48%)	81 (16.33%)	97 (19.56%)	136 (27.42%)	86 (17.34%)	33 (6.65%)
Limiting alcohol intake	18 (3.63%)	31 (6.25%)	42 (8.47%)	25 (5.04%)	48 (9.68%)	84 (16.94%)	248 (50.00%)
Physical activity	27 (5.44%)	67 (13.51%)	102 (20.56%)	63 (12.70%)	81 (16.33%)	80 (16.13%)	76 (15.32%)
Not smoking	31 (6.25%)	8 (1.61%)	11 (2.22%)	5 (1.01%)	13 (2.62%)	28 (5.65%)	400 (80.65%)

Number = 496 for all variables.

Table S13. Frequency of adherence to preventative measures

Preventative measure	Never	Almost never	Sometimes	About half of the time	Most of the time	Almost always	Always
Stay home	1 (0.20%)	4 (0.81%)	10 (2.02%)	5 (1.01%)	27 (5.44%)	126 (25.40%)	323 (65.12%)
Keep 2m distance	0 (0%)	2 (0.40%)	6 (1.21%)	3 (0.60%)	52 (10.48%)	189 (38.10%)	244 (49.19%)
Do not meet others	24 (4.84%)	5 (1.01%)	6 (1.21%)	5 (1.01%)	23 (4.64%)	64 (12.90%)	369 (74.40%)
Wash hands 20+ seconds	0 (0%)	7 (1.41%)	20 (4.03%)	25 (5.04%)	71 (14.31%)	137 (27.62%)	236 (47.58%)
Cover mouth when coughing	0 (0%)	6 (1.21%)	17 (3.43%)	13 (2.62%)	50 (10.08%)	131 (26.41%)	279 (56.25%)
Do not touch eyes/nose/mouth	8 (1.61%)	22 (4.44 %)	39 (7.86%)	38 (7.66%)	127 (25.60%)	145 (29.23%)	117 (23.59%)

Number = 496 for all variables.
